# The association between farmers’ participation in herd health programmes and their behaviour concerning treatment of mild clinical mastitis

**DOI:** 10.1186/1751-0147-54-62

**Published:** 2012-11-02

**Authors:** Ann-Kristina Lind, Peter T Thomsen, Simo Rintakoski, Mari N Espetvedt, Cecilia Wolff, Hans Houe

**Affiliations:** 1 Department of Large Animal Sciences, Faculty of Health and Medical Sciences, University of Copenhagen, Grønnegårdsvej 8, Denmark; 2 Department of Animal Science, Aarhus University, Blichers Allé 20, Tjele, DK-8830, Denmark; 3 Department of Veterinary Biosciences, University of Helsinki, P.O. Box 66, Helsinki, FI-00014, Finland; 4 Department of Production Animal Clinical Science, Norwegian School of Veterinary Science, P.O. Box 8146 Dep, Oslo, NO-0033, Norway; 5 Department of Clinical Sciences, Swedish University of Agricultural Sciences, P.O. Box 7054, Uppsala, SE-750 07, Sweden

**Keywords:** Farmer behaviour, Theory of Planned Behaviour, Mild clinical mastitis, Herd health programme

## Abstract

**Background:**

In Denmark, it has recently become mandatory for all dairy farmers with more than 100 cows to sign up for a herd health programme. Three herd health programmes are available. These differ in a number of aspects, including the frequency of veterinary visits and the farmer’s access to prescription drugs. The objective of this study was to investigate whether dairy farmers’ behavioural intentions, i.e. to call a veterinarian or start medical treatment on the day that they detect a cow with mild clinical mastitis (MCM), are different depending on the type of herd health programme.

**Methods:**

A questionnaire survey based on the Theory of Planned Behaviour (TPB) was conducted. TPB proposes that a person’s behavioural intention is strongly correlated with his or her actual behaviour. Three behavioural factors determine the behavioural intention: attitude, subjective norm and perceived behavioural control. Each of these factors is decided by a set of beliefs, each of which in turn is weighted by an evaluation: 1) the expected outcomes of performing the behaviour, 2) what a person believes that others think of the behaviour, and 3) the person’s perceived power to influence the behaviour.

A set of statements about the treatment of MCM based on interviews with 38 dairy farmers were identified initially. The statements were rephrased as questions and the resulting questionnaire was distributed to 400 randomly selected Danish dairy farmers who use the two most restrictive herd health programmes, either Core or Module1, and to all 669 farmers with the least restrictive herd health programme, Module2. The association between intention and the herd health programme was modelled using logistic regression.

**Results:**

The farmers with the Module2 herd health programme had a significantly higher behavioural intention to perform the behaviour, when compared to farmers with a more restrictive herd health programme (OR = 2.1, p < 0.0001).

**Conclusion:**

Danish dairy farmers who participate in Module2 herd health programme had a higher intention to treat cases of MCM, compared to farmers who participate in a more restrictive herd health programme in which the veterinarian initiates treatments.

## Introduction

In Denmark disease recording for dairy cattle is mandatory. The practice of herd health advisory visits by veterinarians in Denmark was introduced by legislation in 1995 [1]. For all herds with at least 100 adult cattle it became mandatory the first of July 2010 to sign up for a herd health programme, whereas this remains voluntary for smaller herds. Dairy farmers can choose to sign up for one of three different herd health programmes: 1) Core, 2) Module1, 3) Module2 [2]. The Core programme allows the farmer to perform re-treatments on young stock, while the herd veterinarian initiates treatments and re-treats adult cows if needed. In the Module1 programme, the veterinarian initiates the treatment but the farmer is allowed to re-treat adult cows. In the Module2 programme, the farmer is allowed both to initiate treatment and to perform re-treatments on the cows. Thus, depending on the herd health programme, farmers have different options for access to treatment for their animals [2].

The decision-making and behaviours shown by farmers are likely to be influenced by many different factors e.g. severity of symptoms and the value of the cow (factors like milk yield, parity or pregnancy status), some of which are complex, context-related and contain elements that are difficult to quantify [3]. Vaarst et al. [3] found that the decision making differed the most for mild symptoms of mastitis compared to severe symptoms. It has been hypothesised that a decision about treatment is influenced by how ‘easily’ the farmer can initiate individual treatments. It is therefore of interest to investigate whether intentions to treat cases of mild clinical mastitis (MCM) differ according to the herd health programme in operation. MCM was defined according to the International Dairy Federation (IDF) [4], observable abnormalities in milk, generally clots or flakes; little or no signs of swelling of the udder or affected quarter; no other signs that the cow is unwell, no fever, and normal appetite. Investigation of differences in the intentions of dairy farmers to contact a veterinarian on the day they detect signs of MCM in a lactating dairy cow has been carried out recently as part of a larger study in the Nordic countries (Denmark, Finland, Norway and Sweden) that is validating the dairy cow disease databases. Contacting the veterinarian or to initiate treatment was identified as the first and necessary, step in the process that leads to a disease event being recorded in the Nordic countries dairy disease databases [5,6], and the current study was carried out in parallel with the study carried out in the Nordic countries. In recent years there has been an increased focus on the influence of human behaviour and attitude on disease incidence and management, especially in relation to udder health [7-9]. Our objective was to investigate whether farmers who signed up for the Module2 herd health programme and farmers having either ‘Core’, ‘Module1’ or no herd health programme had different behavioural intentions regarding the treatment of MCM.

## Materials and methods

### Target and study populations

In a questionnaire survey ModuleVet herds were sampled randomly from three different sub-populations whereas all Module2 herds were included. The target population for this study was Danish dairy farmers with an average herd size of 15 cows or more. There were two study populations of interest: dairy farmers in Denmark with either herd health programme in which they can initiate treatment themselves (Module2) or herd health programmes in which they need to call a veterinarian to diagnose and initiate treatment (‘None’, ‘Core’ and ‘Module1’), hereafter referred to as ModuleVet.

### Obtaining data on farmers’ behaviour

**Figure 1:**
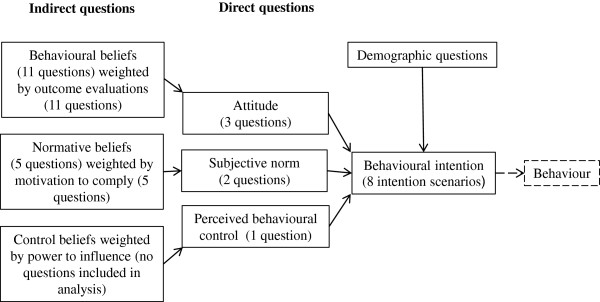
**A schematic presentation of the Theory of Planned Behaviour [after Ajzen and Fishbein,**[12]**].** Attitude, subjective norm and perceived behavioural control were measured with general, direct questions. Attitude and subjective norm were also measured with specific questions concerning underlying beliefs for attitude and subjective norm, respectively, and a corresponding weighting question.

To obtain data on the behaviour of farmers, a questionnaire based on the social psychology model ‘Theory of Planned Behaviour’ (TPB) was used [10,11]. The model is an extension of the earlier ‘Theory of Reasoned Action’ (TORA) [12]. Social science can help us understand and predict human behaviour. TPB explores a person’s intention to perform a specific behaviour and the three underlying factors that determine behavioural intention. In other words, it is possible to use behavioural intention as a proxy for actual behaviour. This intention is in turn explained by three theoretical constructs: attitude, subjective norm and perceived behavioural control. Underlying beliefs are also investigated, the focus being on beliefs about: 1) the expected outcomes of performing the behaviour, 2) what the person believes others think of the behaviour, and 3) the person’s power to influence his or her behaviour. Each belief is weighted by an evaluation (Figure 1). Figure 1 shows a schematic presentation of the structure of the TPB questionnaire. The behavioural intention is a proxy of the farmers’ behaviour and measured by eight intention scenarios. To measure the farmers’ attitude and subjective norm towards the behaviour, both direct and indirect questions were included in the questionnaire. The use of the terms direct and indirect questions describes the difference between general, broad questions about the behaviour in contrast to more specific, narrow questions. All indirect questions were developed from face-to-face interviews carried out with 38 farmers as has been done by others [6]. The face-to-face interviews supplied only a few statements that potentially could be related to perceived behavioural control. Therefore the perceived behaviour control question was only measured by one direct question.

Two different behaviours were investigated in this study, depending on which herd health programme the farmers participated in. The behaviour studied among ModuleVet farmers was the intention “*to contact a veterinarian for a visit on the same day as a dairy cow with MCM is detected”*. For farmers signed up for the ‘Module2’ herd health programme, the behaviour to study was the intention “*to start medical treatment on the same day as a dairy cow with MCM is detected”*.

### Construction of the questionnaires

For development of the questionnaire, the methodology described by Francis et al. [13] was followed. The behaviour to be investigated was defined by the elements of Target, Action, Context and Time (TACt). More specifically, and following the TACt principle, the ModuleVet behaviour to be investigated was to “contact a veterinarian (A) on the same day (t) as a dairy cow with MCM (T) is detected (C)”; for Module2 the behaviour to be investigated was to “start medical treatment (A) on the same day (t) as a dairy cow with MCM (T) is detected (C)”. The first section of the questionnaire addressed the demographics of the farmer, and was followed by a second section that contained eight case scenarios to measure behavioural intention. The third section contained questions to address the farmer’s attitude, which was measured by three direct questions and 11 behavioural beliefs weighted by their 11 corresponding outcome evaluations. The fourth section addressed the subjective norm, measured by two direct questions and five questions on normative beliefs weighted by five questions assessing the motivations to comply. The fifth section included questions to assess the perceived behavioural control; from this section only one direct question was used in the analysis (Figure 1). Finally, the sixth section provided an opportunity for the respondent to supply comments.

The questionnaires for ModuleVet (see Espetvedt et al.[6]) and Module2 (see Appendix 1) were identical, with the exception that the questions in the latter concerned the behaviour ‘to start medical treatment the same day’ instead of ‘contacting a veterinarian for a visit the same day’. Intention can be measured in different ways, but intention simulations are recommended for measuring complex behaviours [13]. Simulations were done with the presentation of eight case scenarios, which described different situations involving cases of MCM in a lactating dairy cow.

### Sampling method

Among the farmers in the ModuleVet herd health programme (approximately 4000), a simple random sample of 400 was obtained from survey selection at random (SAS Institute, Inc., Cary, NC, USA). Further, all 669 farmers who participated in the Module2 herd health programme were selected. According to Garforth (2009, personal communication) a sample size of a minimum of 100 completed questionnaires is recommended for a TPB study and a response rate of 30% could be expected [14], therefor a random sample of 400 was made.

### Data collection and control

In April 2010, the questionnaires were distributed to the sampled herds. After three weeks, a reminder, together with a new questionnaire, was distributed to all non-responders. Farmers to whom the questionnaire could not be delivered by the postal service, were removed from the study (n=4). Following this, the survey response rate after one reminder was 64% (256 returned questionnaires) for ModuleVet and 60% (403 returned questionnaires) for Module2. In total, 659 questionnaires were available for initial data control.

All the returned questionnaires were scanned electronically (Eyes & Hands forms™, RedSoft®). Subsequently, the questionnaires were proofread in order to check for any scanning mistakes, incorrect data entry, and to identify any unreadable handwriting that the scanning machine had difficulty scanning. Data from both types of questionnaire were combined into a common data file using SAS software (Version 9.2, SAS Institute Inc., Cary, NC, USA), which was used for all data management and analysis. Histograms were plotted for all questions to identify observations with illegal values and questions with little variation in the answers. Observations with > 25% missing or > 50% neutral answers (n=13) were considered to represent poorly completed questionnaires, and were deleted. Thereafter, the final sample consisted of 646 responders.

As a quality control for the questions, the internal consistency and reliability of the test scores of direct items for both attitude and subjective norm were measured by Cronbach’s alpha [15]. The internal consistency was 0.76 and 0.79 for attitude and 0.84 and 0.80 for subjective norm for ModuleVet and Module2, respectively. Cronbach’s alpha can be considered to be acceptable for values > 0.6 [13]. Cronbach [15] stated that “a high alpha is to be desired but a test need not approach a perfect scale to be interpretable”; however, according to DeVellis [16], Cronbach’s alpha can be considered to be acceptable for values between 0.60 and 0.70. Higher Cronbach’s alpha values are reported as ‘respectable’ or ‘very good’.

A dichotomised variable for behavioural intention was created from the answers to the eight case scenarios: high intention, > 50% ‘yes’ answers, and low intention, ≤ 50% ‘yes’ answers. The direct attitudes were re-categorised from a 1–7 Likert scale to a scale of low, medium and high attitudes on which 1–3 represented low attitude, 4 medium attitudes and 5–7 high attitude. Direct subjective norm and perceived behavioural control were scored on Likert scales from 1–7 with words in each end of the scale being semantic opposites, for example ‘important-unimportant’, ‘agree-disagree’ [17]. The demographics questions were all class variables, except for age and herd size which were on a continuous scale, but these were re-categorised into categorical variables defined according to quartiles for presentation of the descriptive statistics.

Underlying the attitude are a person’s beliefs of a certain outcome if the behaviour is carried out. These beliefs are weighted by how desirable the outcome is. Underlying the subjective norm is the normative belief items, meaning the social pressure to carry out the behaviour. These normative beliefs are weighted by the strength of the motivation to comply with this social pressure. The indirect attitude and the normative beliefs items were multiplied by their corresponding evaluation item and the products were summarised to give composite (1) indirect attitude and (2) subjective norm class variable measures:

1) Attitude α Σb_i_ e_i_, the strength of each belief (b) of the i^th^ item weighted by the evaluation (e).

2) Subjective Norm α Σn_i_ m_i_, the strength of each normative belief (n) of the i^th^ item weighted by the motivation to comply (m).

To further evaluate the questionnaire design, Spearman rank correlation coefficients between the direct and indirect composites were calculated to test the internal consistency (Figure 1). The correlation coefficients were found to be in the range 0.33 to 0.39 for all four correlations. According to Ajzen and Fishbein [12], a Spearman rank correlation coefficient > 0.3 is acceptable.

### Data analysis

Descriptive statistics were calculated for ModuleVet and Module2 herd health programmes. To test the hypothesis of different behavioural intentions for ModuleVet and Module2 farmers, a logistic regression model was used (PROC LOGISTIC, SAS version 9.2). First all explanatory variables were screened one by one. All variables with a p-value < 0.2 in this univariable model were included in the final multivariable analysis where after model refinement was performed by backward stepwise elimination of main effects. Herd size was tested in the model both as a categorical and as a continuous variable. Two-way interactions were tested for all possible combinations of explanatory variables. The final multivariable model (1) was identified:

(1) $$y_{i}=\alpha +B_{i}+C_{i}+D_{i}+E_{i}$$

where y_i_ is the high intention of the i^th^ item, α is the intercept, B is the type of herd health programme (Module2 or ModuleVet), C is the direct attitude (low, medium, high) and D is age (continuous). To correct the model for confounding, changes in the estimates were checked when removing variables. A variable was considered to be a confounder if the change in the odds ratio was over 20% as the chosen cut-off when adding and removing the variable [13]. The goodness of fit was tested using the chi-square/degrees of freedom.

Exact confidence intervals (CI) at 95% significance level were calculated [18] for all parameters that were used for evaluating how representative the study herds were compared to all national herds. The study herds were compared with the national herds in the milk recording scheme with respect to herd size, %-fat, %-protein and mean energy corrected milk yield per cow and classified as significantly different if the CIs were non-overlapping. Summary statistics on all Danish dairy herds originated from the Danish Cattle Federation [19].

## Results

### Descriptive statistics

The median scores for behavioural intention, direct attitude, subjective norm and perceived behavioural control can be seen in Table 1. The median behavioural intention scores, related to categories of demographic variables, were in general higher for Module2 compared with ModuleVet (Table 2). Females and males had the same median behavioural intention score if they participated in the same herd health programme. The age distribution of farmers is presented as a categorical variable in Table 2, and 63% of the farmers in ModuleVet were over 45 years of age compared with 36% for Module2. Module2 farmers had the same behavioural intention score regardless of age, whereas farmers younger than 45 years of age with the ModuleVet herd health programme had lower behavioural intention compared with those above 45 years of age in this programme. The frequencies of different herd sizes varied between the two types of herd health programmes. The farms with Module2 herd health programme were larger than those with ModuleVet, but the median behavioural intention score was similar for all four herd size categories for Module2, and was lowest for herds with ModuleVet that had a herd size in the range 100 to 200 dairy cows. No organic farmers participated in Module2 because the regulations for organic farms do not allow treatments to be initiated by other than the veterinarian (Table 2). There was a tendency that Module2 farmers more frequently took milk samples and sent them for analysis without first consulting a veterinarian than ModuleVet farmers, among whom 62% stated that they never take a milk sample and send it for analysis without first consulting a veterinarian (Table 2).

**Table 1:** Descriptive statistics for behavioural intention, direct attitude, direct subjective norm and direct perceived control from a Theory of Planned Behaviour survey in Denmark involving farmers with two different categories of herd health programmes

	**Possible range**	**ModuleVet n=252 median (Q1, Q3)**	**Module2 n=392 median (Q1, Q3)**
Behavioural intention score	0 to 1	0.50 (0.25, 0.63)	0.63 (0.38, 0.75)
Direct attitude	1 to 7	5.00 (3.33, 6.00)	5.33 (4.00, 6.00)
Direct subjective norm	1 to 7	4.50 (3.00, 6.00)	4.50 (3.00, 6.00)
Direct perceived behavioural control	1 to 7	6.50 (5.50, 7.00)	6.00 (5.50, 7.00)

The behaviour of interest was “contacting the veterinarian for a visit the same day as noting a case of mild clinical mastitis in a lactating dairy cow” for the herd health programme where the veterinarian initiates treatment (ModuleVet), and “start medical treatment on the same day as noting a case of mild clinical mastitis in a lactating dairy cow” for the herd health programme in which farmers may initiate medical treatment themselves (Module2).

n = the number of observations. Q1 = first quartile. Q3 = third quartile.

**Table 2:** **The frequency (%) and the median intention (Q1;Q3)**^**1**^**score of answers to demographic questions, for the herd health programme in which the veterinarian initiates medical treatment (ModuleVet) and the herd health programme in which the farmer may initiate medical treatment (Module2)**

**Question**	**Answer**	**ModuleVet**		**Module2**	
		**Freq (%)**	**Median intention (Q1;Q3)**	**Freq (%)**	**Median intention (Q1;Q3)**
Gender of the respondent	Male	240 (95.6)	0.5 (0.29;0.63)	356 (92.7)	0.63 (0.38;0.75)
	Female	11 (4.4)	0.5 (0.25;0.63)	28 (7.3)	0.63 (0.44;0.88)
Age^2^	≤40	62 (25.0)	0.38 (0.25;0.63)	163 (42.7)	0.63 (0.38;0.75)
	>40-≤45	31 (12.5)	0.38 (0.13;0.63)	80 (20.9)	0.63 (0.38;0.88)
	>45-≤50	60 (24.2)	0.5 (0.33;0.63)	56 (14.7)	0.63 (0.38;0.63)
	> 50	95 (38.3)	0.5 (0.38;0.75)	83 (21.7)	0.63 (0.38;0.75)
Herd size^1^	>15-≤100	113 (45.9)	0.5 (0.25;0.75)	24 (6.4)	0.56 (0.31;0.69)
	>100-≤150	74 (30.1)	0.46 (0.25;0.5)	98 (26.0)	0.63 (0.38;0.75)
	>150-≤200	31 (12.6)	0.38 (0.13;0.63)	100 (26.5)	0.63 (0.38;0.75)
	>200	28 (11.4)	0.5 (0.13;0.63)	155 (41.1)	0.63 (0.38;0.75)
Percentage of the household income that comes from the dairy business	<25%	10 (4.1)	0.56 (0.5;0.63)	28 (7.5)	0.56 (0.31;0.75)
	25%	6 (2.5)	0.68 (0.5;0.88)	9 (2.4)	0.63 (0.5;0.75)
	50%	45 (18.5)	0.37 (0.25;0.63)	51 (13.6)	0.63 (0.38;0.75)
	75%	91 (37.8)	0.5 (0.25;0.63)	137 (36.5)	0.5 (0.38;0.71)
	100%	91 (37.5)	0.5 (0.25;0.75)	150 (40.0)	0.63 (0.5;0.75)
Type of milking system	Pipeline milking	77 (30.7)	0.5 (0.25;0.75)	12 (3.4)	0.63 (0.38;0.75)
	Robot	59 (23.5)	0.38 (0.25;0.5)	115 (29.7)	0.5 (0.38;0.71)
	Milking parlour	104 (41.4)	0.46 (0.19;0.63)	204 (52.7)	0.63 (0.38;0.75)
	Milking carrousel	7 (2.8)	0.5 (0.25;0.63)	41 (10.6)	0.63 (0.5;0.88)
	Other	4 (1.6)	0.38 (0.06;0.68)	14 (3.6)	0.44 (0.13;0.63
Stall type	Tie stall	69 (27.4)	0.5 (0.38;0.75)	11 (2.9)	0.63 (0.38;0.88)
	Free stall with deep bedding	16 (6.4)	0.5 (0.13;0.56)	24 (6.2)	0.56 (0.25:0.63)
	Free stall with cubicles	158 (63.0)	0.38 (0.25;0.63)	345 (89.4)	0. 3 (0.38;0.75)
	Combination tie stall/free stall	8 (3.2)	0.56 (0.38;0.69)	6 (1.6)	0.63 (0.63;0.63)
How often a milk sample is taken by the farmer and sent for analysis without first consulting a veterinarian	Never	157 (62.6)	0.5 (0.25;0.63)	85 (22.0)	0.63 (0.38;0.75)
	Sometimes	57 (22.7)	0.5 (0.25;0.63)	158 (40.1)	0.63 (0.38;0.75)
	Fairly often	18 (7.2)	0.5 (0.38;0.75)	57 (14.6)	0.5 (0.38;0.75)
	Very frequently	10 (4.0)	0.44 (0.38;0.63)	52 (13.3)	0.63 (0.38;0.63)
	Always	9 (3.6)	0.38 (0.25;0.5)	37 (9.5)	0.63 (0.38;0.75)
Herd type	Conventional	215 (86.0)	0.5 (0.25;0.63)	391 (100)	0.63 (0.38;0.75)
	Organic	35 (14.0)	0.25 (0.0;0.5)	0 (0)	-
The farmer’s own classification of clinical mastitis incidence in the herd the last year	Very high	3 (1.2)	0.5 (0.25;0.88)	22 (5.7)	0.63 (0.5,0.88)
	High	35 (13.9)	0.5 (0.25;0.63)	70 (18.0)	0.63 (0.38,0.75)
	Medium	108 (43.0)	0.5 (0.38;0.63)	200 (51.7)	0.63 (0.38,0.75)
	Low	77 (30.7)	0.38 (0.13;0.63)	86 (22.4)	0.63 (0.29,0.75)
	Very low	28 (11.2)	0.38 (0.0;0.63)	9 (2.3)	0.63 (0.13,0.63)
Any non-family employees working with the dairy cows	Yes	126 (49.8)	0.5 (0.25;0.63)	323 (82.9)	0.63 (0.38;0.75)
	No	125 (50.2)	0.5 (0.25;0.63)	66 (17.1)	0.63 (0.38;0.75)

^1^ Q1 = first quartile. Q3 = third quartile.

^2^ The age of the farmer and herd size were reorganised into four categories, defined according to the quartiles within the category for both ModuleVet and Module2.

### Behavioural intention

Among the ModuleVet farmers, 37% (92 out of 252) had a high intention to call a veterinarian on the same day as they noticed a cow with MCM, compared with 54% (210 out of 390) of Module2 farmers who intended to initiate treatment (Table 3). Further, in the multivariable regression model, the intention was significantly higher for Module2 farmers to start medical treatment than for ModuleVet farmers to contact veterinarian (OR = 2.1, p < 0.0001) (Table 4). The variables that influenced the behavioural intention were direct attitude, age and herd health programme; these variables were included in the final model (Table 4). Farmers with high attitude had 7.6 times higher odds of having high intention, when compared to farmers with low attitude. Age affected the intention, but the effect was minor because the odds ratio was only 1.2 for every 10 years increase in age.

**Table 3:** The number of farmers in each behaviour intention category which were created by dichotomizing the behavioural intention into either ‘high’ or ‘low’ intention for the herd health programme, in which the veterinarian initiates medical treatment (ModuleVet) and for the herd health programme, in which the farmer may initiate medical treatment (Module2), respectively

**Intention**	**ModuleVet**	**Module2**	**Total**
High^1^	92	210	302
Low^2^	160	180	340
Total	252	390	642

^1^ High intention > 50% yes answers.

^2^ Low intention ≤ 50% yes answers.

**Table 4:** Results of the final multivariable logistic regression model to assess the relationship between high intention and predictor variables using odds ratios with 95% Wald confidence intervals (95% CI)

**Variable**	**Class**	**Odds ratio**	**95% CI**	**p-value**
Herd health programme^1^	Module2	2.1	1.5 – 3.1	<0.0001
	ModuleVet	Ref.	.	.
Direct attitude	High	7.6	4.9 –11.7	0.0001
	Medium	1.9	0.9 – 4.0	
	Low	Ref.		
Age	10 years^2^	1.2	1.0 –1.5	0.0260

^1^ Herd health programme, in which veterinarians initiate treatment (ModuleVet) and the herd health programme, in which farmers may initiate treatment (Module2).

^2^ for an increase of every 10 years.

Two-way interactions were tested in the model but were not statistically significant. The final multivariable model (1) was identified, and all the remaining effects were statistically significant at p-value < 0.05 (Table 4). The overall fit of the model was tested using the chi-square (χ^2^ = 1.02) and values close to 1 indicates a good model fit.

### Representativeness of study herds

The Module2 study herds were significantly different from the average statistics of all Danish dairy herds. All production parameters were higher for Module2 compared to all Danish Dairy herds. Herd size and mean energy corrected milk per cow were significantly different between ModuleVet and Module2 whereas %-fat and %-protein was not significantly different between the two herd health programmes. The %-fat and %-protein for ModuleVet and Module2 were both significantly higher than the average for all Danish dairy herds (Table 5). There were only minor differences in the mean energy corrected milk yield per cow (kg) for the study herds compared to all Danish dairy herds.

**Table 5:** For comparison of representativeness of the study herds, descriptive statistics (means (95% confidence intervals)) are shown

	**ModuleVet**	**Module2**	**Danish dairy herds**^**1**^
Herd size	121.5 (115;128)	210 (200;217)	127^2^
%-fat	4.37 (4:31;4.42)	4.36 (4.32;4;41)	4.26
%-protein	3.47 (3.45;3:50)	3.46 (3.44;3:48)	3.41
Mean energy corrected milk yield per cow (kg)	8795.5 (8661;8929)	9282.8 (9182;9378)	8922

The study herds were compared with the average of all Danish dairy herds participating in milk recording in 2008.

^1^ Data on all Danish dairy herds are from Danish Cattle Federation [19].

^2^ The herd size in 2010 [20].

## Discussion

The results of this study showed that farmers in Module2, i.e. farmers that are allowed to initiate treatment themselves without prior examination of the cow by a veterinarian, had significantly higher behavioural intention to start medical treatment as the intention of ModuleVet farmers to contact a veterinarian. The case scenarios that measured behavioural intention were written in such a way that it was not obvious, under Danish conditions, if the case of MCM should be treated with prescription drugs the same day or not. Whether it is good to have a high or a low intention can be discussed. Not all MCM cases require medical treatment according to the behaviour intention found in this study; for example ModuleVet farmers would treat 50% of the MCM cases. ModuleVet farmers had a median intention score of 0.50, and this would have been 1.00 if the belief was that all the MCM behavioural intention scenarios presented should be treated medically on the same day as they were discovered.

The type of herd health programme and the attitude of the farmer described most of the variation in intention between the ModuleVet and Module2 farmers. The Module2 farmers had an odds ratio of 2.1 compared to ModuleVet farmers, which indicates that the Module2 farmers would be more likely to initiate treatment of a case of MCM on the same day as noticing it, whereas ModuleVet farmers might be more inclined to wait for a day or two before contacting the veterinarian for a visit, or may choose not to call the veterinarian at all. In a recent study, it was found that Denmark had the highest farmer detected treatment of clinical mastitis when compared with the other Nordic countries [21]. These results are in line with the farmers’ relatively high intention score, 0.63 for Module2 and 0.50 for ModuleVet, to initiate treatment/call a veterinarian on the same day as they noticed a cow with MCM, when compared with findings in other Nordic countries. Compared to results from the TPB study carried out in the Nordic countries, Norwegian farmers had a behavioural intention score of 0.5, comparable to DK ModuleVet farmers, in contrast to 0.38 and 0.0 for Swedish and Finnish farmers, respectively [5,6]. Finland has no intention to contact the veterinarian. However, in Finland it is more common to take a milk sample before contacting a veterinarian for a visit, and this explains the low intention found [6].

The differences in behavioural intention observed between farmers with the different herd health programmes in this study could be explained by variations in the cost of treatment. ModuleVet is associated with the more direct cost of a veterinarian’s fee, incurred when the veterinarian is contacted for a visit to a cow with MCM. Module2 farmers have medicine available at the farm, which means that the cost is more indirect because they have already paid for the scheduled visits by the veterinarian. It would be interesting to study the reasons why farmers choose the Module2 programme, for example if they already before entering had a more positive attitude to treatment of MCM than farmers who retain their Module1 or Core herd health programme. Or a speculation is that a higher behavioural intention to initiate treatment is a consequence of the easier access to, for example, antibiotics, that Module2 farmers have available on farm. Farmers with high attitude had 7.6 times higher odds of having high behavioural intention compared with farmers with a low attitude; this means that these farmers have a positive attitude towards medical treatment.

The median herd size for Module2 was larger than that for ModuleVet. This was expected, since the economic incentives to participate in Module2 are higher for large herds. On the other hand, there was no clear tendency for behavioural intention to differ among herds of different sizes with the same type of herd health programme.

Most farmers who participate in ModuleVet state that they never or only sometimes take milk samples without contacting a veterinarian. This is logical, because they would need to arrange a veterinary visit to have the cow treated with prescription drugs. However, there was a tendency for farmers who often take milk samples without first contacting a veterinarian also to state a lower behavioural intention to contact a veterinarian on the same day as detecting a case of MCM. This could be explained by, that these farmers have a strategy in which they await the results from a milk sample before a veterinarian is contacted. Interestingly, Module2 farmers stated the same behavioural intention to initiate treatment on the same day regardless of how often they take milk samples without first contacting a veterinarian. Farmers in Module2 are required to take milk samples if the cow is treated with any antibiotics other than penicillin. Whether farmers in Module2 initiate treatment of cases of MCM with such antibiotics depends on their herd health programme. This might be one explanation for the variation among Module2 farmers in their tendency to take milk samples without contacting a veterinarian first.

It could be expected that participating farmers did not initially have the same understanding of a MCM case. To overcome this issue great emphasis was placed on explaining the definition, both during elicitation interviews, in the questionnaire and ensuring that case scenarios in the questionnaire conformed to the IDF definition [4]. In a study by Andersen et al. [22] it was shown that veterinary specialists did not agree on a mastitis definition based on concrete examples. However small differences in understanding of this definition would not be expected to make the results of this study invalid as the emphasis was on assessing differing treatment thresholds. If there was a different understanding of MCM this was likely random within the two groups. There is no evidence that Module2 farmers all thought of moderate/severe mastitis when answering questions about attitude, subjective norm and perceived behavioural control, and that ModuleVet farmers all thought of mild cases of clinical mastitis.

The questionnaire for the Module2 farmers was developed from the final ModuleVet questionnaire, which was developed to address differences in behavioural intention in the Nordic countries. According to Francis et al. [13] it is preferred that 75% of the most important statements obtained judged by the frequency that these statements are mentioned, from the elicitation study are represented in the final questionnaire. We included around 50% of the most important statements to avoid an excessively long questionnaire, which is one problem with the TPB approach. TPB-based questionnaires tend to be fairly complex; the questions asked need to be worded very precisely and can often be seen as repetitious [23]. A long and complex questionnaire often results in a low response rate. Other authors have regarded a response rate of 20–30% as satisfactory [14], the response rates of 60% and 64% obtained in this study may be considered high. Still, selection bias, which may have affected the results, cannot be excluded. It is likely that there is an overrepresentation of farmers interested in treatment of mild clinical mastitis. Even if some farmers are more interested in mastitis, it may not mean that variation in behaviour either towards or against deciding upon treatment of mastitis is more uniform than in the rest of the population. The study herds were compared with the average of all Danish dairy herds and significance differences were obtained between Module2 study herds and all Danish dairy herds and even between ModuleVet and all Danish dairy herds. The study herds had in general higher production parameters. All farmers participating in the Module2 herd health programme were included in the study. It is not surprising that these herds differ from the average of all Danish dairy herds according to the production parameters since the choice of Module2 herd health programme may be influenced by both the herd size and the health status on farm. For ModuleVet there were no significant difference in the herd size and mean energy corrected milk yield per cow compared to the average of all Danish herds.

## Conclusion

There were significant differences in behavioural intention between farmers depending on which of the two herd health programmes they participated in. The differences in intention were explained by the underlying attitude, the age of the farmer and by the herd health programme that the farmer participated in.

## Competing interests

The authors declare that they have no competing interests.

## Authors' contributions

All authors participated in the study design. AL was responsible for data collection and performed the statistical analysis, interpreted the data and drafted the manuscript. HH, PT and SR participated in the statistical analyses. All authors revised the manuscript, and have read and approved the final manuscript.
